# Effects of depth on reef fish communities: Insights of a “deep refuge hypothesis” from Southwestern Atlantic reefs

**DOI:** 10.1371/journal.pone.0203072

**Published:** 2018-09-26

**Authors:** Pedro Henrique Cipresso Pereira, Cláudio Henrique Macedo, José de Anchieta C. C. Nunes, Laura Fernandes de Barros Marangoni, Adalto Bianchini

**Affiliations:** 1 Universidade Federal de Pernambuco (UFPE), Departamento de Oceanografia, Recife (PE), Brazil; 2 Projeto Conservação Recifal (Reef Conservation Project), Recife, Pernambuco, Brazil; 3 Laboratório de Ecologia Bentônica (LEB), Universidade Federal da Bahia, Salvador-Bahia, Brazil; 4 Programa de Pós-Graduação em Oceanografia Biológica, Instituto de Oceanografia, Universidade Federal do Rio Grande, Rio Grande, Rio Grande do Sul, Brazil; 5 Instituto de Ciências Biológicas, Universidade Federal do Rio Grande, Rio Grande, Rio Grande do Sul, Brazil; University of California Santa Cruz, UNITED STATES

## Abstract

Deeper reefs are often considered to be less susceptible to local and global disturbances, such as overfishing, pollution and climate change, compared to shallow reefs and therefore could act as refugia for shallow water species. Hence, the interest on deeper reefs has happened at a time when shallow reefs are undergoing unprecedented changes. Here we investigated the hypothesis that fish community differed from shallow to deeper reefs due to factors apart from habitat structure and quality and therefore discuss for the first-time insights of a “deep refuge hypothesis” from Brazilian reefs. We collected data on fish community, benthic community and physiological conditions of two coral species on shallow (< 6 m) and deep reefs (> 25 m). No significant difference on substratum composition was observed comparing sites and depths. Additionally, physiological data on corals also showed similar oxidative status and growth conditions when comparing the two-coral species in shallow and deep reefs. Conversely, our study demonstrated strong differences on reef fish communities in terms of abundance, species richness, trophic groups, size classes and groups of interest when comparing shallow and deeper reefs. Fish abundance was 2-fold higher and species richness was up to 70% higher on deeper reefs. Also, a significant difference was observed comparing trophic groups of reef fish. Macrocarnivore, Mobile invertebrate feeders, Planktivores, Sessile Invertebrates Feeders and Roving Herbivores were more abundant on deeper reefs. On the other hand, Territorialist Herbivores almost exclusively dominated shallow reefs. Strong differences were also observed comparing the abundance of reef fish groups of interest and their respective size classes between shallow and deeper reefs. Ornamental, Great Herbivores and Groupers showed clear differences, with higher abundances being observed in deeper reefs. Considering size classes, larger individuals (> 15 cm) of Great Herbivores, Groupers and Snapper were uniquely recorded at deeper reefs. Additionally, individuals with > 30 cm were recorded almost exclusively on deeper reefs for all the analyzed groups of interest. Our findings suggest that fishing pressure on the target species may be attenuated on deeper reefs, and these regions may therefore be considered as areas of refuge from shallow water impacts. Therefore, the likely potential for deeper reefs protect species from natural or anthropogenic disturbances increases the attention of marine conservation planning and resource management on including deeper reefs in protected areas.

## Introduction

Coral reefs are among the most diverse and productive ecosystems on earth [1, 2]. They play important economic, ecological and social roles such as coastal protection, maintenance of ocean ecological processes and climate regulation [3, 4, 5]. However, coral reefs are in intense decline due to several local and global disturbances such as climate change, overfishing, coral bleaching, predator outbreaks and biological invasion[2, 6, 7];). These disturbances can affect not only the structure and composition of corals and fish communities but also key ecological processes, such as herbivory and recruitment, and therefore the maintenance of ecosystem functioning ([7,8].

Depth is known to influence many factors on reef ecosystems for both coral and reef fish communities, influencing the structure of coral reef communities mainly due to light attenuation, changes in water temperature and resource availability [9, 10, 11]. For instance, depth influences coral distribution, composition and physiology [12, 13, 14]. It has been currently suggested that some coral species exhibit distinct and sometimes opposing physiological adaptations due to low light attenuation; [10, 15]. However, it is still unclear if differences in depth could influence the ecophysiology of coral species (e.g. bleaching susceptibility) and few studies have attempted to understand this relationship so far (e.g. [16]). Additionally, reef fish communities are also strongly influenced by depth with effects on abundance, species richness, size classes, fish trophic guilds and habitat specialization [17, 18, 19, 20, 21]. Different patterns of fish community have been historically found for coral reefs around the world. However, studies assessing the structure of coral reef communities have been strongly focused on shallow reefs and only in the last decades researches have endeavoured to explore deeper and mesophotic coral reefs on the South Atlantic Ocean [22, 23, 24, 25].

Deeper reefs are currently considered to be less susceptible to local and global disturbances, such as overfishing, pollution and climate change, compared to shallow reefs. Thus, they may act as refuges and sources of propagules for shallow threatened reefs (the “deep reef refugia” hypothesis; [16, 26]). The interdependence between shallow and deeper reefs (via larval dispersal or juvenile/adult migration) has intrigued marine ecologists and is a central question on studies focused on deeper reefs [27, 28, 29]. Many coral reef fish, especially commercial species such as groupers and parrotfishes demonstrate a large depth distribution and use to be often recorded on both shallow and deeper reefs [30]. In these context, certain fishing methods performed on coral reefs, particularly breath-hold spearfishing, have obvious depth limitations and deeper reefs could be “safer” compared to shallow habitat. Thus, it is therefore assumed that a proportion of the fish population can obtain refuge in deeper water; [31]. Hence, multiple approaches integrating knowledge on different ecological groups and incorporating field and laboratory analyses may represent important tools for better understanding the deep refuge hypothesis. Conversely, it is worth mentioning that ecological process driving differences between shallow and deep coral reef communities could be related to other variables such as cross-shelf coral reef distribution patterns. Many large-bodied and commercial fish species such as groupers and snappers present evident cross-shelf migration patterns (i.e. recruit in shallow reefs and migrate to deeper habitats where they reproduce) described worldwide [32, 33]) and in Brazilian coast [34]. Brazilian reefs comprise the unique coral reef ecosystems on South Atlantic Oceans, with high endemism rates, and therefore a good model for testing hypothesis related to the deep refuge hypothesis. Brazilian coral reefs are approximately extended 3,000 km along the Brazilian coast, disconnected from the Caribbean Sea by a semi-permeable geographic barrier [35]. Recent studies have described the composition and community structure of deeper reefs in Brazil [25, 36, 37] However, the deep refugee hypothesis has been poorly studied on Brazilian reefs [25] and preliminary data on our deeper study sites suggests that fish populations could be buffered from shallow waters impacts [38].

The present study aims to test the effects of depth on reef fish communities analyzing abundance, species richness, size classes, trophic guild and groups of interest. In order to correlate fish community data with habitat features we accessed benthic community data by comparing shallow (<6 m) and deeper reefs (>25 m) in Brazilian reefs. Lastly, cellular diagnostic parameters (also known as biomarkers) have been applied in two important scleractinian coral species in Brazilian reefs, *Montastraea cavernosa* and *Siderastrea stellata*. Our goal was to assess the cellular/physiological condition of corals according to depth. Thus, ecologically relevant biomarkers related to photo-physiological traits (Chl *a* content), bleaching susceptibility (LPO levels) and calcification (Ca-ATPase activity) were used to assess the health of the two coral species aforementioned. Use of biomarkers enables the identification of stressors or environmentally changing conditions based on an understanding of processes at cellular level [39] and therefore infer on habitat suitability for fish communities. Specifically, we investigated the hypothesis that fish community differed at different depths due to factors apart from habitat structure and quality, and therefore discuss insights of a “deep refuge hypothesis” from Brazilian reefs.

## Materials and methods

### Study area

**Fig 1:**
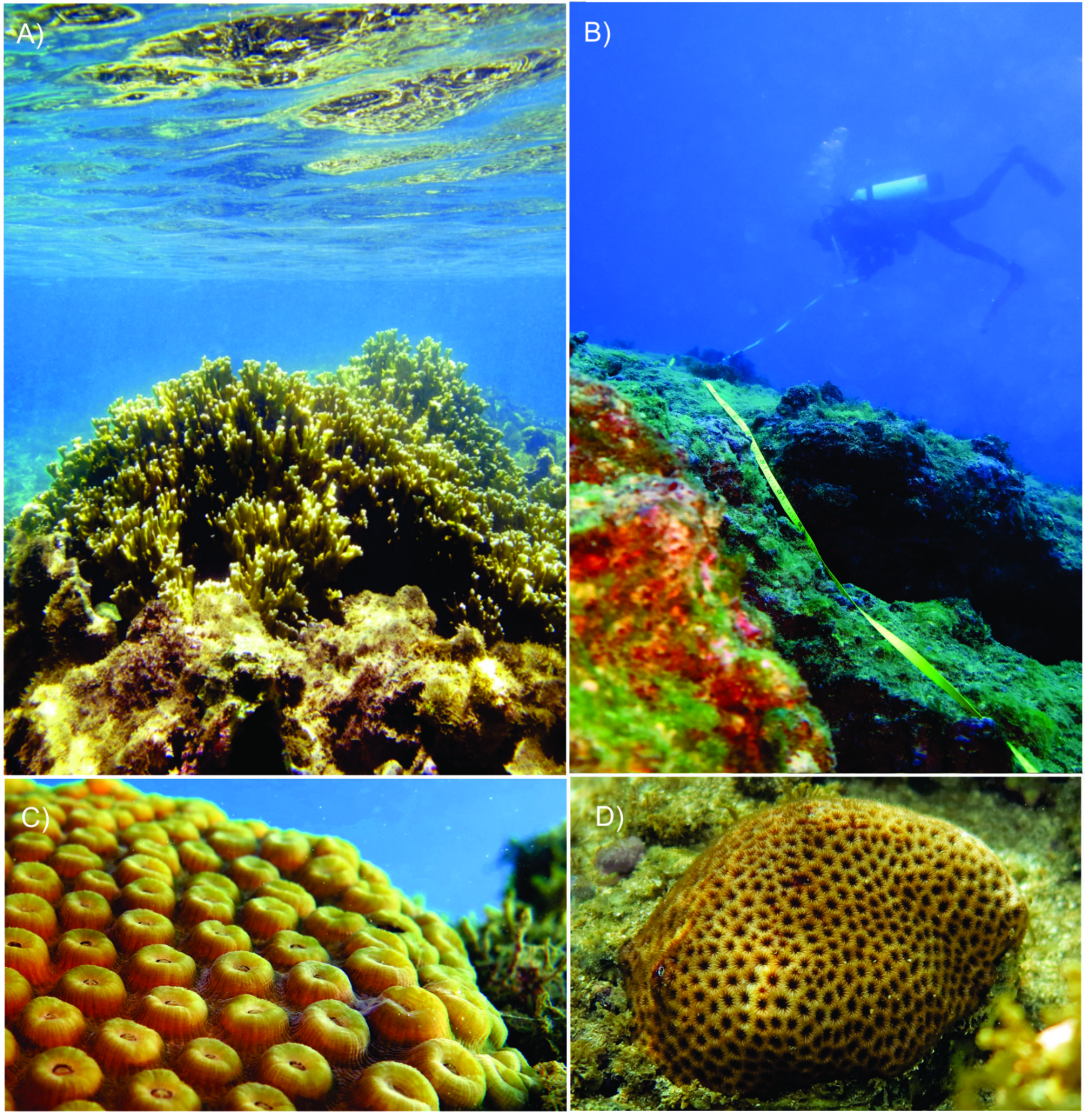
Shallow (A) and deep (B) reefs and studied coral species *Montastraea cavernosa* (C) and *Siderastrea stellata* (D). Photos–Pedro Pereira”.

The present study was conducted during 2015–2016 in four distinct areas aiming a comparison between shallow and deep reefs in northeastern Brazil under Brazilian permit SISBIO n-54029. Shallow areas had an average depth of approximately 4 m: Pirambú (8° 45'31.65 "S 35° 5'8.01" W) and Northern Pirambú (8° 44'32.97 "S 35° 4'50.61" W). On the other hand, Carapitanga (8° 49'35.59 "S 35° 2'43.16" W) and Carapitanga do Norte (8° 49'13.58 "S and 35° 2'21.60" W) were the deep reefs. They are reefs more distant from the main land (around 8 km) and had a depth ranging from 25 to 30 m (Fig 1, also see [38] for site description).

### Benthic community

To measure the coverage and categorization of the substratum (*e*.*g*. benthic community), the Point Intercept Transect (PIT) method was used. This method is most commonly used because of its efficiency and fast data acquisition. It also provides good estimates of coverage of benthic communities [40]. This method consists in positioning of a transect of 20 m, in which every 0.5 m the substrate component was annotated, adding 40 points per track, totalling 40 points per transect. A total of 12 transects were performed per site (Shallow 1, Shallow 2, Deep 1 and Deep 2) with a total of 48 transects randomly distributed. For identification, the substrate was categorized into: (1) Epilithic Algae Matrix (EAM); (2) Hard coral; (3) Macroalgae; (4) Coralline Algae; (5) Zoanthids; (6) Sponge; (7) Bared Rock, and (8) Sand.

In order to produce benthic composition percentage analyses, data were mathematically (arcsine square root) transformed [41]. The arcsine square root transformation has been used for long time as a standard procedure when analyzing proportional data in ecology, with applications in data sets containing binomial and non-binomial response variables [42]. Multivariate analysis was also applied for benthic community data comparing shallow and deep sites. A principal component analysis (PCA) was performed after a centered log-ratio (clr) data transformation and a matrix using euclidean distance for PCA. PCA was performed using the software Primer V6 & PERMANOVA+ [43].

### Biomarkers approach for corals

#### Chlorophyll a (Chl a) quantification

Coral bleaching (as a decrease in photosynthetic pigments in the coral host tissue) is considered a potential physiological response that can be used to assess a diversity of stressors [44]. Also, acclimation of the holobiont to low-light intensity can be achieved by change in the photo-physiological traits of the symbionts, such as changes in pigment (e.g. Chl a) concentrations [45]. Chl *a* concentration in the holobiont was quantified following the method described by Schimidt [46], with some modifications. Briefly, small pieces (~0.5 cm^2^) of the collected corals were cut, placed in Eppendorf-type tubes (2 mL) and sonicated (Sonaer Ultrasonics, Farmingdale, NY, USA) on ice using Milli-Q water until the tissue was detached from the skeleton. For Chl *a* extraction, 18 μl of the sample homogenate was vortexed with 382 μl of cold ethanol (≥99.5), boiled for 5 min at 80°C and kept at 4°C for 24 h. After the extraction step, the sample homogenate was centrifuged and had its absorbance read at 750 and 665 nm using a microplate reader (ELx-800, Biotek, Winooski, VT, USA). In all steps, sample homogenates were protected from light. Chl *a* concentration was normalized considering the amount of total proteins present in the sample homogenates [47]. Data were expressed as ng Chl *a* μg protein^-1^. Total proteins content in the sample homogenates was determined using a commercial reagent kit based on the Bradford assay (Sigma-Aldrich, St. Louis, MO, USA).

#### Sample preparation for biochemical biomarkers analyses

Samples were prepared for lipid peroxidation level and Ca-ATPase activity measurements following the procedures described by Downs [39], with some modifications. Briefly, samples of the holobiont were ground in liquid nitrogen, divided into aliquots (150–200 mg) and sonicated (Sonaer Ultrasonics, Farmingdale, NY, USA) on ice using the specific homogenization buffers (1:2, w/v) required for analysis of each biomarker, as described below. Sample homogenates were centrifuged (13,000 *g*) at 4°C for 10 min. The intermediary phase was collected and immediately used for analyses.

#### Lipid peroxidation (LPO)

Biomarkers related to oxidative stress can be used to evaluate reef environment health by quantifying the level of damage to biomolecules (*e*.*g*. lipids and DNA). Therefore they can be good predictors of bleaching/mortality in coral reef organisms [48, 49, 50, 51]. Lipid peroxidation (LPO) measurement was performed using the fluorimetric method described [52], which quantifies oxidative stress by measuring the peroxidative damage to lipids induced by free radicals [52]. For both species, samples were homogenized in 1.15% KCl solution containing 35 μM butlylated hydroxytoluene (BHT). The peroxidative damage to lipids was quantified through the reaction between the thiobarbituric acid (TBA) and malondialdehyde (MDA), a byproduct of lipid peroxidation. The reaction generates a chromogen, which is measured by spectrofluorometry. Fluorescence (excitation: 515 nm; emission: 553 nm) was measured using a fluorometer (Victor 2, Perkin Elmer, Waltham, MA, USA). Data were normalized considering the amount of total proteins in the sample homogenates. Data were expressed as nmol MDA mg protein^-1^. Total protein content in sample homogenates was determined using a commercial reagent kit based on the Bradford assay (Sigma-Aldrich, St. Louis, MO, USA).

#### Ca-ATPase activity

Calcification represents a crucial process for the growth of scleractinian corals, and therefore of vital importance in the structuring and functioning of coral reefs [53]. In the present study, Ca-ATPase activity was used as a biomarker of this process, since Ca-ATPase is recognized as a key-enzyme in the calcification process in scleractinian corals. Indeed, it has been used as a good indicator of growth rates in calcifying reef organisms [50, 51, 54, 55, 56]. Ca-ATPase activity was measured under optimized substrate concentrations for each species, following a modified protocol originally developed by Chan [57]. For both species, sample homogenates were prepared using a buffer solution containing 500 mM sucrose, 1 mM dithiothreitol (DTT), and 1 mM phenylmethylsulfonyl fluoride (PMSF), and 100 mM trizma hydrochloride (Tris-HCl) at pH 7.6. Samples were centrifuged (10,000 *g*) at 4°C for 20 min prior analysis. The working buffer solution contained 189 mM NaCl, 1 mM ouabain and 20 mM Tris-HCl at pH 7.6. Optimized substrates concentrations for *M*. *cavernosa* were 10 mM CaCl_2_ and 3 mM ATP, while for *S*. *stellata* they were 3 mM CaCl_2_ and 20 mM ATP. Sample homogenates were incubated with 250 μl of reaction medium (working buffer + substrates) and placed into a water bath at 30°C for 30 min. The reaction was stopped by placing the mixture on ice for 10 min. Ca-ATPase activity was measured based on the amount of inorganic phosphate (Pi) released in the reaction medium, considering that the enzyme converts ATP to ADP with consequent release of Pi. Pi concentration in the reaction mixture was measured using the commercial reagent kit "Fosfato" (Doles, Goiânia, Goiás, Brazil), which is based on the colorimetric method described by Fiske [58]. Measurements were performed at 630 nm using the microplate reader (ELx-800, Biotek, Winooski, VT, USA). Data were normalized considering the amount of total proteins in the sample homogenates. They were expressed as mM Pi mg protein^-1^ min^-1^. Total protein content in sample homogenates was determined using a commercial reagent kit based on the Bradford assay (Sigma-Aldrich, St. Louis, MO, USA).

### Data presentation and statistical analyses

Data were expressed as mean ± standard error of the mean (SE). Data on biochemical biomarkers for *M*. *carvernosa* and *S*. *stellata*, from shallow and deeper reefs, were compared using a mixed-model analysis of variance (ANOVA). Depth was used as a fixed factor while collection sites were considered as random factors nested within depth. If indicated, ANOVA were followed by the post hoc Student-Newman-Keuls (SNK) multiple comparison test. In all cases, ANOVA assumptions (data, normality and homogeneity of variances) were previously verified using the Shapiro-Wilk and Levene´s tests, respectively. Data were log-transformed when necessary. In all cases, significance level adopted was 95% (α = 0.05).

### Reef fish community

Reef fish community was also accessed by belt transects in the same sites of benthic composition. This method consists in the use of 20 m long transects where all fish individuals and their sizes are recorded (Visual Census) (2.5 m to the right and 2.5 m to the left of the track) (Brock, 1982). Fishes were classified into seven size categories, at intervals of 5 cm (0–5, 6–10, 11–15, 16–20, 21–25, 26–30, <31). Species were also classified according to their trophic category: (1) Macrocarnivore (MCAR); (2) Mobile invertebrates feeders (MIF); (3) Omnivores (OMN); (4) Piscivores (PIS); (5) Planktivores (PLK); (6) Sessile Invertebrates Feeders (SIF); (7) Roving Herbivores (ROVH); and (8) Territorialist Herbivores (TERH). The species trophic categorization followed the information available in the literature on dietary habits [59, 60]. Groups of interest were also standardized aiming to better understand differences on fish community when comparing shallow and deeper reef. Groups of interest included Ornamental, Great Herbivores, Groupers, Snappers and Pelagics. Information available in the literature was also used to classify fish into groups of interest [59, 60].

Univariate and multivariate analyses were applied on reef fish community data. WILCOXON [61] test was used for the factor “Depth”. This nonparametric comparison of two groups was used to verify if the population means are different. A permutational multivariate analysis of variance (PERMANOVA) [62] was used to test the null hypothesis. In this case, we tested if the fish community is different between shallow and deeper reefs. Statistical significance of PERMANOVA was tested with 9999 residuals permutations under an unrestricted permutation method and type III (partial) sums of squares. Therefore, a "two-way nested PERMANOVA" was used for the factors "Depth" and "Site nested within Depth. We also used the PERMANOVA approach for the analysis of trophic categories only for the factor “Depth”. The similarity percentage analysis (SIMPER) [63] only for factor “Depth” was used to ascertain which species contributed the most with the dissimilarity comparing shallow and deeper reefs. All multivariate analysis was performed using the software Primer V6 & PERMANOVA+ [43].

The similarity matrix based on Bray Curtis was generated and the multidimensional scaling (MDS) analysis was performed to verify the samples distribution aiming for a better understanding of the differences in depth between the study areas and sites. From this matrix, samples were graphically represented by a non-metric multidimensional scaling (nMDS) ordination [63]. MDS was performed using the software Primer V6 & PERMANOVA+ [43].

## Results

### Benthic community

**Fig 2:**
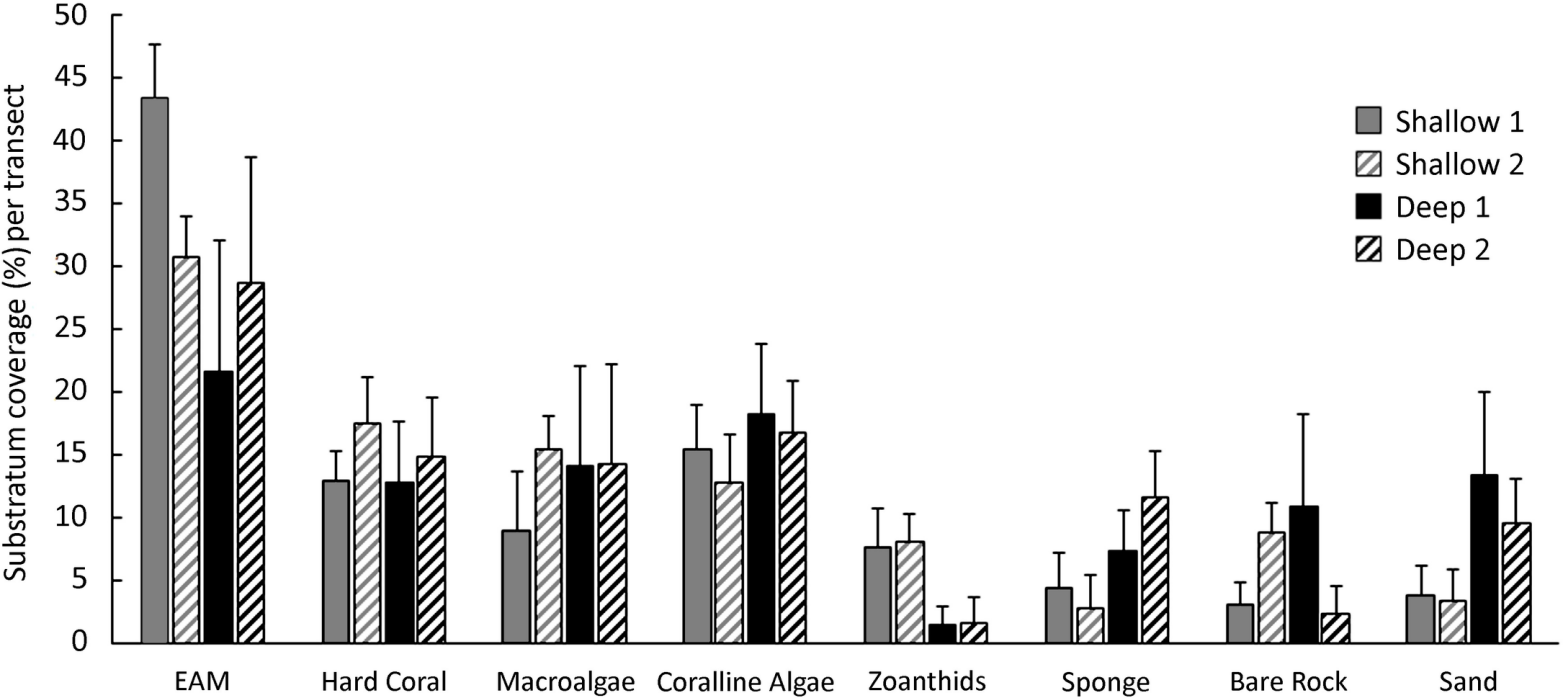
Substratum coverage (mean average ± SD) between shallow and deep reefs in northeastern Brazil.

Ephilitic algae matrix (EAM) was the most abundant substratum category recorded in all studied sites in shallow (shallow 1: 43.43%; shallow 2: 30.77%) and deeper reefs (deep 1: 21.70%; deep 2: 28.76%) (Fig 2). Additionally, hard coral, macroalgae and coralline algae had similar covers among sites. In general, no significant difference was observed regarding substratum coverage between sites and depths (ANOVA; *F* = 224.56; *p* > 0.01).

**Fig 3:**
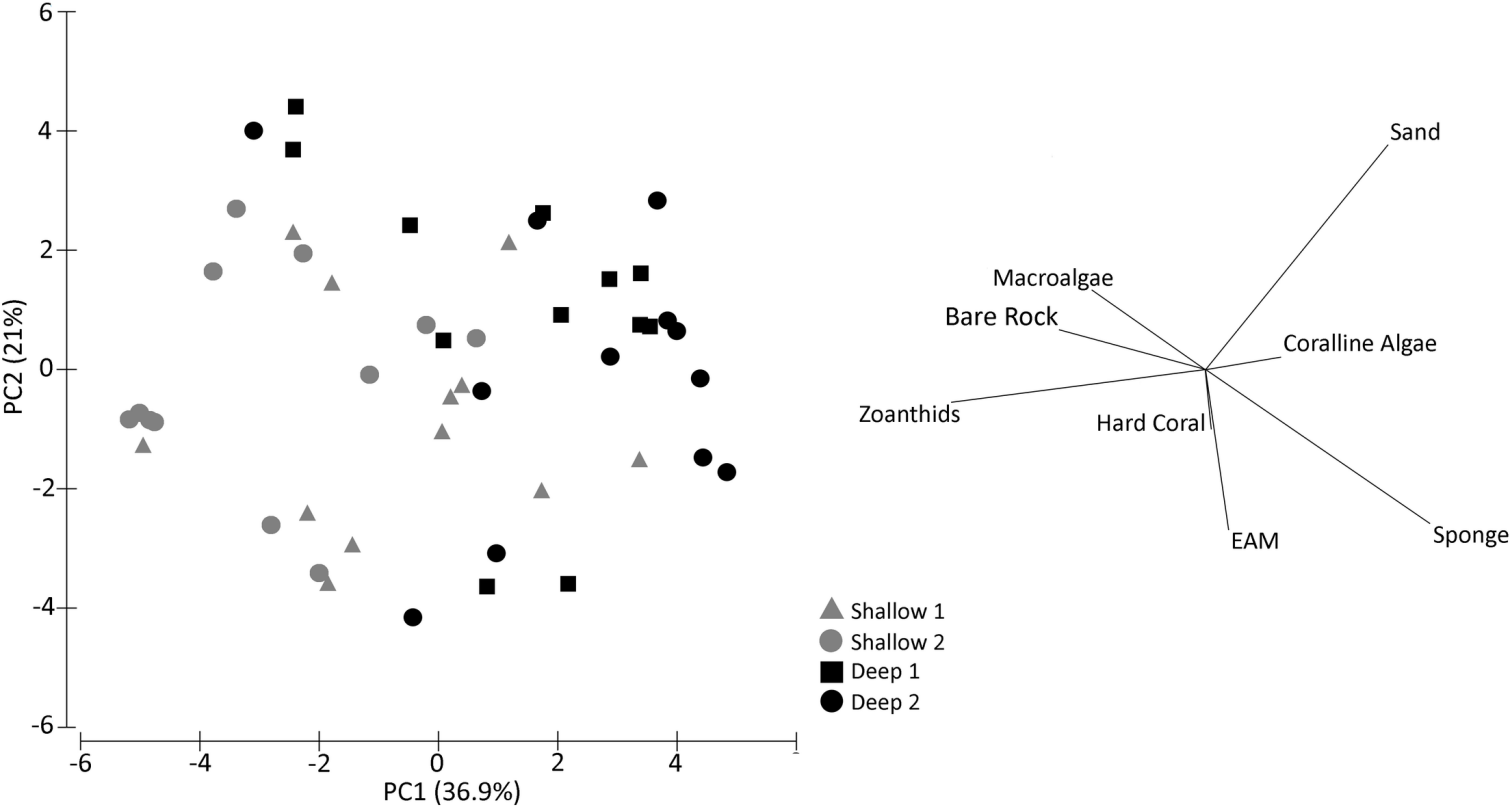
PCA analysis with substratum coverage considering shallow and deep reefs in northeastern Brazil.

PCA analysis showed that PC1 and PC2 explained 36.9% and 21.0% of the data variation respectively. Sponge, sand and coralline algae were more correlated with deeper reefs (Deep 1 and 2). In contrast, bare rocks and zoanthids were more correlated with shallow reefs (Shallow 1 and 2) (Fig 3).

### Biomarkers approach for corals

#### Chlorophyll a (Chl a) content

**Fig 4:**
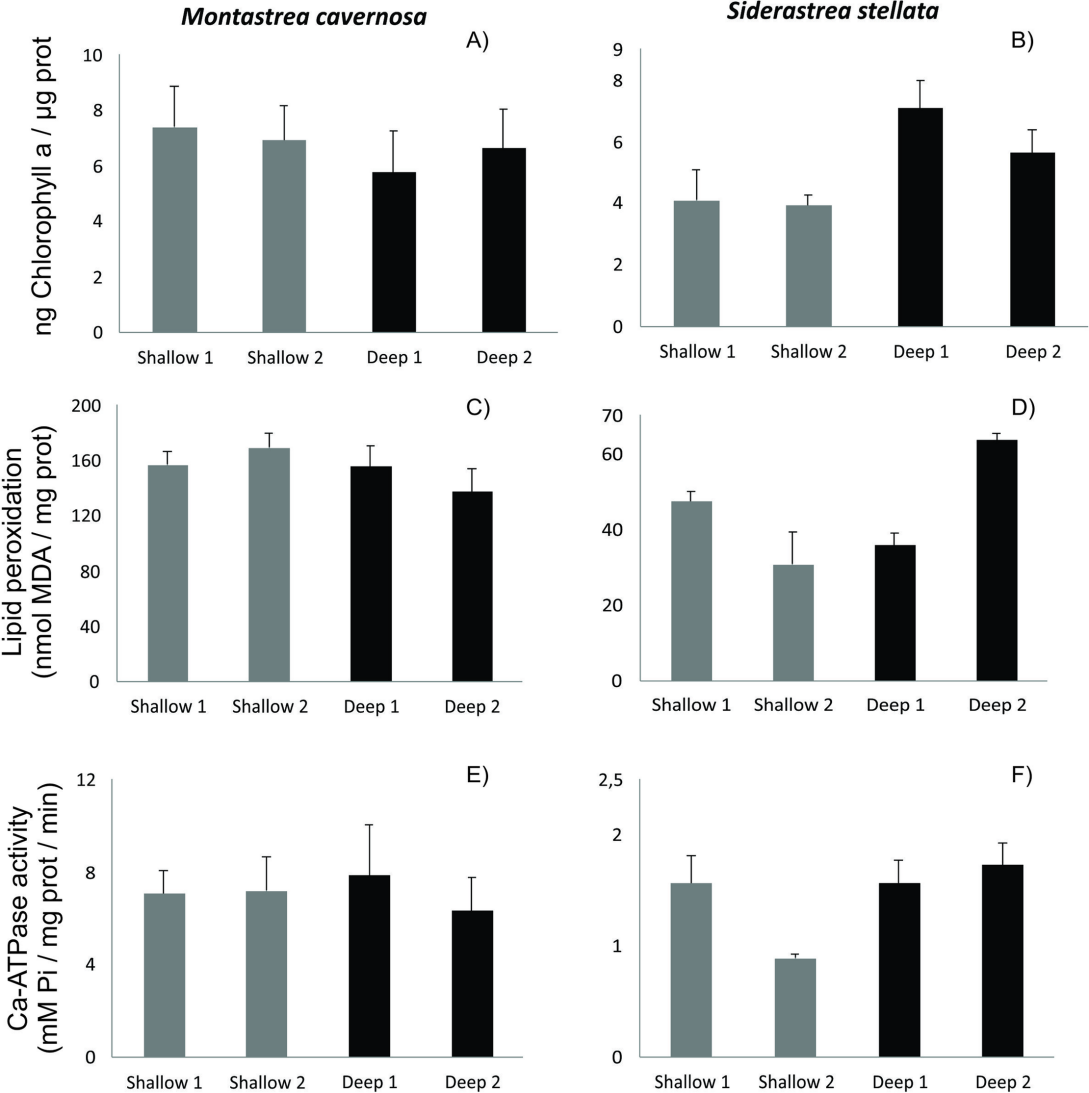
Chlorophyll a (Chl a) concentration, lipid peroxidation (LPO) and Ca-ATPase activity in the corals *Montastraea cavernosa* and *Siderastrea stellata* collected in shallow and deep reefs in northeastern Brazil.

For both corals *M*. *carvernosa* (Fig 4A) and *S*. *stellata* (Fig 4B), there were no significant differences in the holobiont Chl *a* content between sites of collection at the same depth, as well as between shallow and deeper reefs.

#### Lipid peroxidation (LPO)

For the coral *M*. *carvernosa* holobiont, no significant differences in LPO levels were observed between sites of collection at the same depth, as well as between shallow and deeper reefs (Fig 4C). In turn, the coral *S*. *stellata* holobiont showed significant different levels of LPO between sites of collection at both depths. Mean values of LPO were higher (~1.5-fold; p<0.001) at the shallow 1 site when compared to the shallow 2 site. However, mean values of LPO were higher (~1.8-fold; p<0.001) at the deep 1 site when compared to the deep 2 site (Fig 4D).

#### Ca-ATPase activity

In the coral *M*. *carvernosa* holobiont, there were no significant differences in Ca-ATPase activity between sites of collection at the same depth, as well as between the shallow and deep reefs, (Fig 4E). In turn, mean values of Ca-ATPase activity were significantly higher (~1.8-fold; p<0.05) in the coral *S*. *stellata* holobiont at the shallow 1 site when compared to the shallow 2 site. However, no significant differences were observed between the sites of collection in the deep reefs (Fig 4F).

### Reef fish community

**Fig 5:**
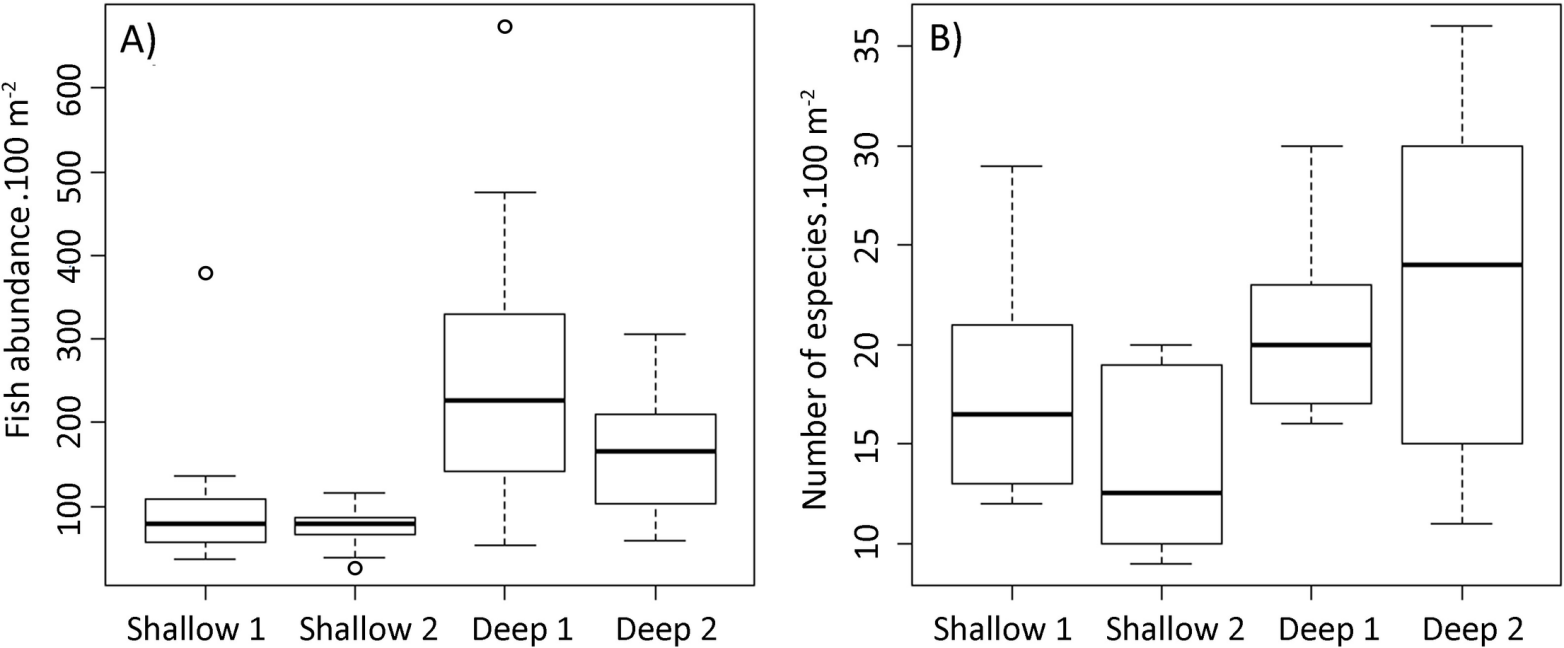
Reef fish abundance (A) and species richness (B) on shallow and deep reefs in northeastern Brazil. Box plots show the interquartile range, with whiskers indicating the respective confidence intervals. The horizontal lines indicate median values and circles represent the outliers of the total length that presented greater variability.

A significant difference in fish abundance was observed comparing depths in the study area (W = 247, p <0.001), with higher abundance (up to 450 individuals/100 m^2^) being observed in deeper sites (Fig 5A). Species richness was also significant different between shallow and deep reefs (W = 191; p < 0.001), with higher values (up to 35 species/100 m^2^) being observed in deeper reefs (Fig 5B). PERMANOVA analysis also revealed significant differences in reef fish communities between depths and sites. When comparisons were made between sites, differences are only seen in the shallow reefs. However, there were no differences between sites in the deep reefs (Table 1).

**Table 1:** Reef fish community results of the two-way nested PERMANOVA with factor “Site” nested within factor “Depth”. Posteriori pair-wise tests results for the factor “Site(Depth)” are also shown.

Factor	df	SS	MS	Pseudo-F	P(perm)	Unique Perms
Depth	1	34604	34604	28.72	**<0.001**	9935
Site(Depth)	2	8646	4323	3.59	**<0.001**	9935
Residuals	68	81921	1204.7			
Total	71	125170				
Pair-wise tests for factor “Site(Depth)”				t	P(perm)	Unique Perms
*Within level “Shallow” of factor “Depth”*						
Pirambu x North Pirambu				2.28	**<0.001**	9931
*Within level “Deep” of factor “Depth”*						
Carapitanga X North Carapitanga				1.25	0.06	9922

**Fig 6:**
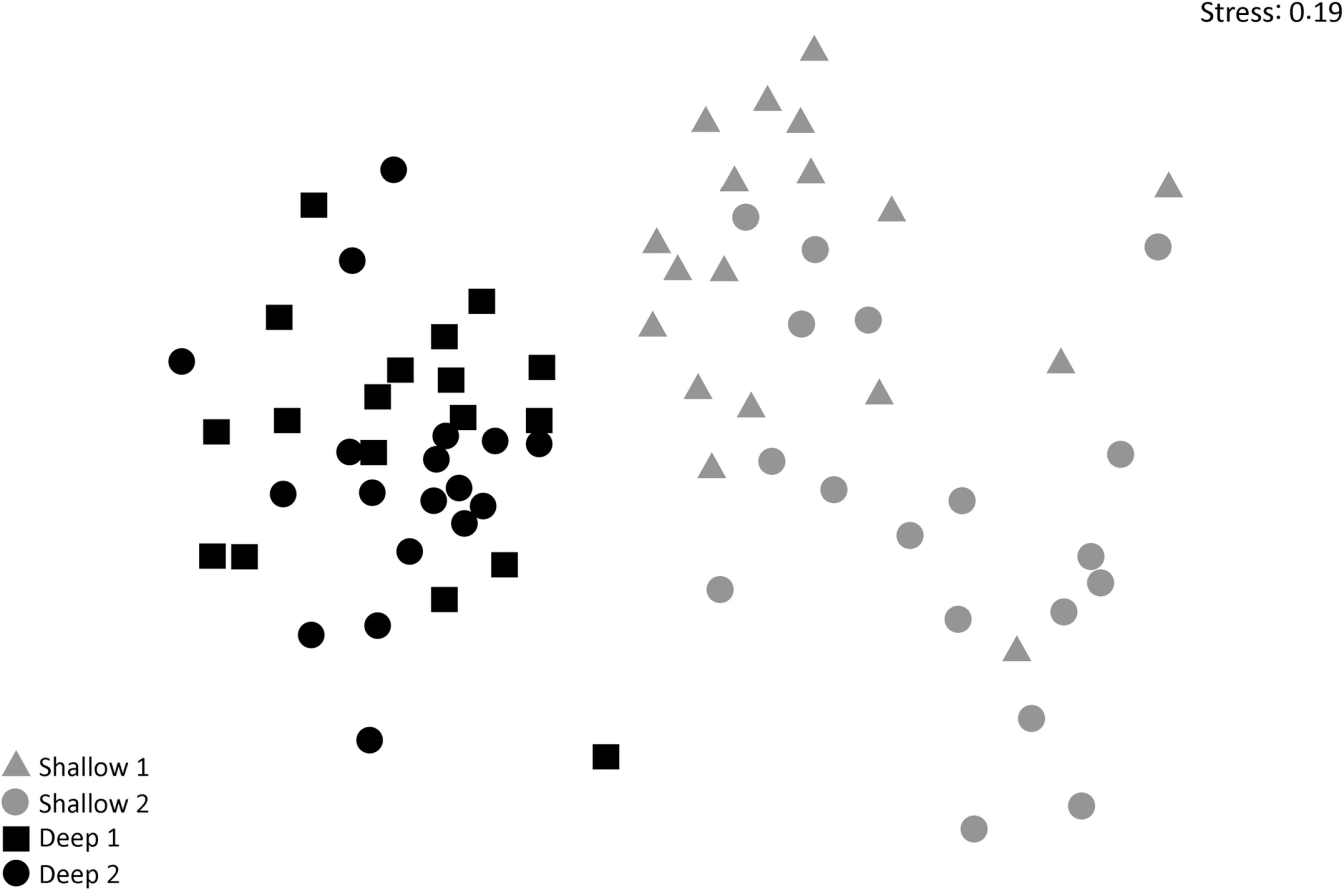
Multidimensional scaling (MDS) comparing reef fish communities in shallow and deep reefs in northeastern Brazil.

With a stress of 0.19 when comparing shallow and deeper reefs, the non-metric multidimensional (MDS) scaling ordination highlights the differences between reef fish communities among habitats. The MDS analysis demonstrated a formation of two distinct groups, with strong distinction between depths (Fig 6).

The SIMPER analysis indicated *Stegastes fuscus*, *Bodianus rufus* and *Gramma braziliensis* as being the fish species that contributed most to the dissimilarity between depths (11.0%, 5.5% and 4.4% respectively) (Table 2). The fish *S*. *fuscus* was the most abundant species in the present study, but its greater occurrence was concentrated in the shallow reefs. On the other hand, *B*. *rufus* and *G*. *braziliensis* were also abundant species, but almost uniquely recorded at deeper reefs (Table 2).

**Table 2:** SIMPER results of dispersion-based weighting data of reef fish species with an overall contribution greater than 50% to the dissimilarity between shallow and deep reefs.

Species	Mean count		Contribution (%)	Cumulative Contribution (%)
	Shallow	Deep		
*S*. *fuscus*	4.62	0.16	11.42	11.42
*B*. *rufus*	0.01	2.33	5.50	16.92
*G*. *brasiliensis*	0.04	1.95	4.45	21.37
*H*. *tricolor*	0.00	1.71	4.31	25.68
*C*. *fulva*	0.44	2.05	4.20	29.87
*A*. *bahianus*	1.29	2.22	4.06	33.93
*S*. *zelindae*	0.29	1.61	3.49	37.42
*S*. *frondosum*	0.14	1.35	3.21	40.62
*H*. *poeyi*	1.43	0.68	3.13	43.76
*H*. *aurolineatum*	0.12	1.28	3.09	46.85
*E*. *figaro*	0.22	1.30	2.76	49.61
*A*. *saxatilis*	0.66	1.10	2.74	52.35

Mean dissimilarity = 79.94

**Fig 7:**
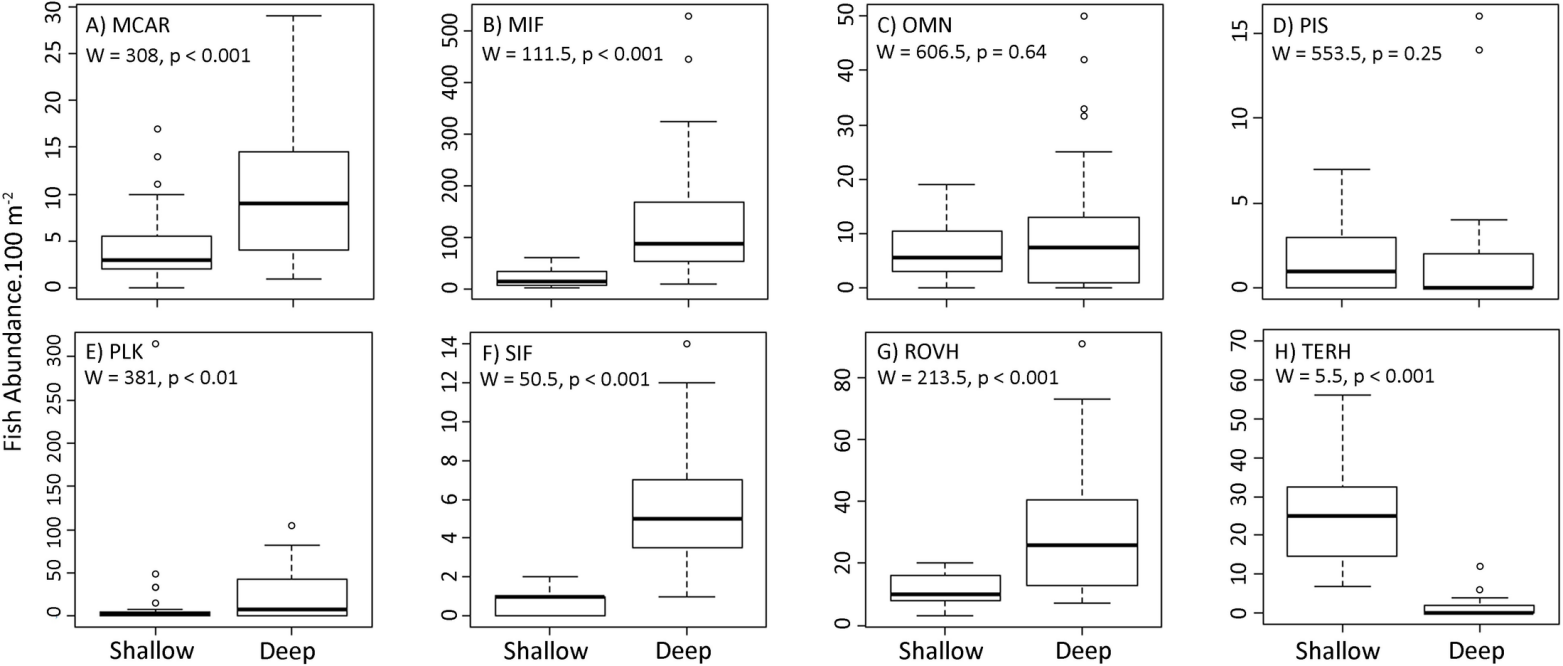
Trophic groups when comparing reef fish assemblages in shallow and deep reefs in northeastern Brazil. Macrocarnivore (MCAR); Mobile invertebrate feeders (MIF); Omnivores (OMN); Piscivores (PIS); Planktivores (PLK); Sessile Invertebrates Feeders (SIF); Roving Herbivores (ROVH) and Territorialist Herbivores (TERH).

Also, a significant difference was observed when comparing reef fish trophic groups (Pseudo-F = 67.52, P (perm) < 0.001) (Fig 7). Macrocarnivore, Mobile Invertebrate Feeders, Planktivores, Sessile Invertebrates Feeders and Roving Herbivores were more abundant in deep reefs. On the other hand, Territorialist Herbivores were almost exclusively found in shallow reefs. Piscivores and Omnivores did not show significant differences between shallow and deep reefs (Fig 7).

**Fig 8:**
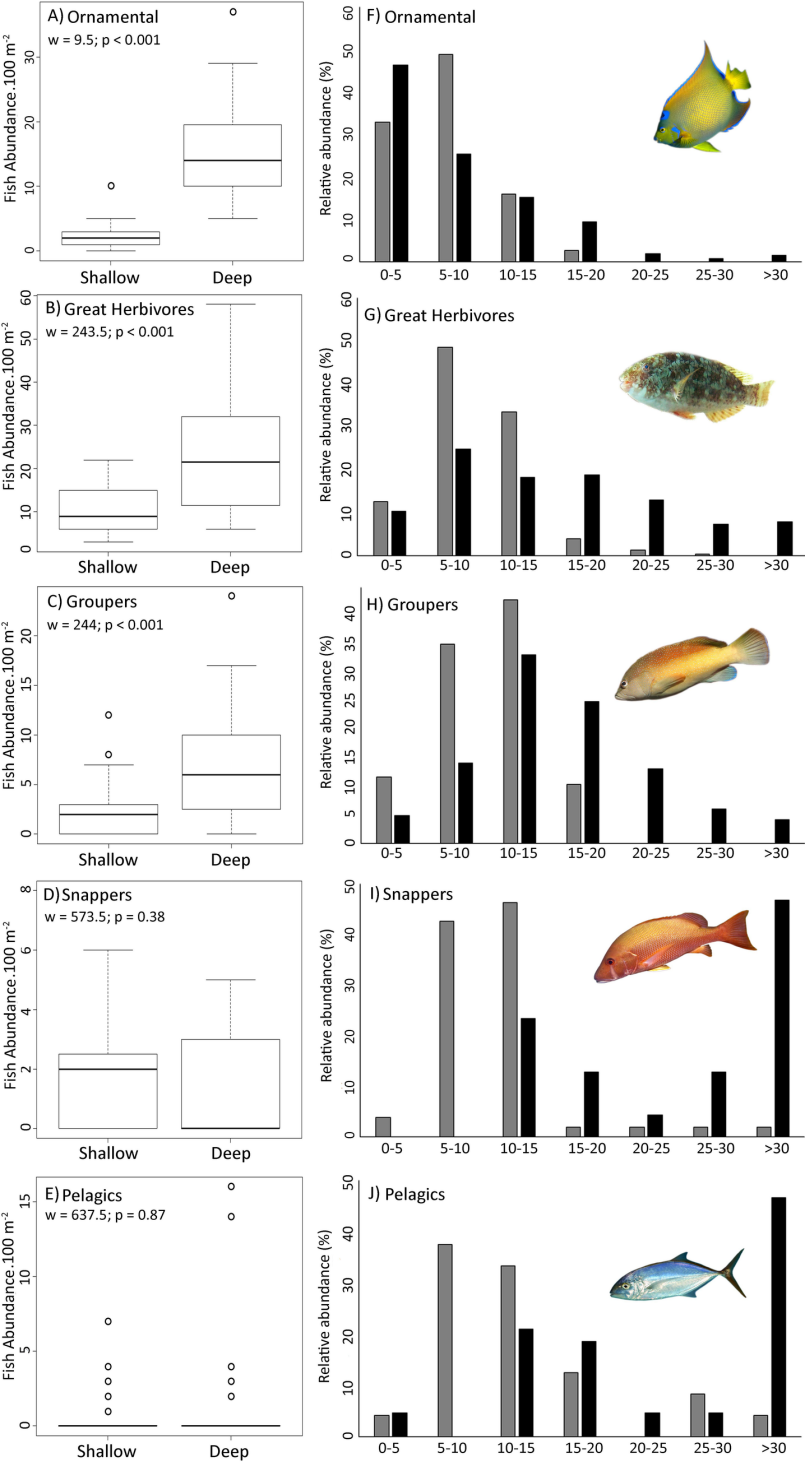
Groups of interest and their respective size classes when comparing reef fish assemblages in shallow and deep reefs in northeastern Brazil. Gray bars = shallow reefs and dark bars = deeper reefs.

Significant differences were also observed when comparing the abundance of groups of interest of reef fish and their respective size classes between shallow and deep reefs (Fig 8). Ornamental, Great Herbivores and Groupers showed clear differences, with higher abundances in deep reefs. In contrast, no significant differences in fish abundance were observed for the Snapper and Pelagics groups when comparing shallow and deep reefs (Fig 8). Considering fish size classes, larger individuals (> 15cm) of Great Herbivores, Groupers and Snapper were uniquely recorded at deep reefs. Additionally, individuals larger than 30 cm were recorded almost exclusively on deep reefs, for all the analyzed groups of interest (Fig 8).

## Discussion

A current increase body of research has analysed the importance of deeper reefs and its relevance for the maintenance of anthropized shallow coral reef communities (see [64] for a review). However, there is a clear lack of information on how this process occurs and if this potential is universal in space and time [65]. Our study demonstrated strong differences in reef fish communities in terms of abundance, species richness, trophic groups, size classes and groups of interest when comparing shallow and deep coral reefs communities from the South Atlantic Ocean. Despite no difference in habitat features and quality (e.g. cellular diagnostic parameters—biomarkers) were observed for two important coral species, fish community was much more abundant and rich on deeper habitats. Larger fishes, ornamental species and species target on spearfishing such as groupers, snappers and pelagics were also much more abundant on deeper reefs. Therefore, these findings suggest a potential for a “deep refuge hypothesis” in South Atlantic coral reefs. On the other hand, recent studies have suggested that distance from the coast might be as important as depth on influencing reef fish communities especially on the SWA continental shelf, which is wide [66]. In the present study, distance from the coast could definitely be an important variable reducing fishing pressure on deeper reefs; however, those variables are difficult to be analyzed independently once they covary.

In the present study, epilithic algae matrix (EAM) was the most abundant substratum category recorded in all studied sites, with up to 40% of relative abundance. As previously recorded by Rosa [36] in depths around 30 m, EAM is the most abundant benthic category in other depth habitats. Additionally, for the Brazilian coast, [37] also cited that turf algae dominated rocky reefs of varying health levels in deeper reefs of the Vitória-Trindade Seamount Chain (VTC). In the present study, subtle differences in benthic communities were observed when comparing depths, such as higher abundance of zoanthids in shallow reefs (up to 10% of relative abundance) and higher abundance of sponge in deeper habitats (up to 15%). However, no significant difference in substratum composition was observed when comparing sites and depths. Strong similarities in benthic communities emphasizes the likely potential to maintain similar fish communities in shallow and deep reefs (Williams et al., 2015); however, this was not the case in the area evaluated in the present study.

Generalist coral species, with wide bathymetric distributions (*e*.*g*. *Stylophora pistillata* and *Seriatopora hystrix*), change their colony morphology in deeper water toward flat shapes with thinner, wider spreading branches in order to decrease self-shading and maximize light capture [67, 68]. Besides morphological changes to compensate lower light levels, corals can harbor different types (clades) of symbionts, which imply modifications in their photo-physiological traits [15, 16]. Also, this increases the acquisition of heterotophic nutrients to compensate lower photosynthate production [15, 16, 69]. In the present study, ecologically relevant biomarkers related to photo-physiological traits (Chl *a* content), bleaching susceptibility (LPO levels) and calcification (Ca-ATPase activity) were used to assess the health of *M*. *carvernosa* and *S*. *stellata*, two important corals species occurring in shallow and deeper reefs in the Brazilian coast. Altogether, biomarker data indicate no significant difference in cellular-physiological conditions in corals occurring in shallow and deeper reefs, thus suggesting similar bleaching susceptibility and thermal stress for both coral species in different habitats. These results emphasize the recent discussion that the refuge potential of deeper reefs is not a universal trend. Indeed, this could strongly vary according to location, depth and coral species [65, 70], and therefore must be further investigated.

To our knowledge, physiological data regarding oxidative status (*e*.*g*. oxidative damage to biomolecules), between corals of the same species, thriving in different depths, are lacking in literature. Regarding the calcification process, [45] reported lower calcification rates in the coral *S*. *pistillata* occurring in depths above 50 m. However, no data on key enzymes involved in this process (*e*.*g*. Ca-ATPase and carbonic anhydrase) are available in literature so far. Our results suggest that there are no differences in coral calcification between depths. In this case, it is important to note that corals occurring in deep waters analysed in the present study occurred in shallower depths than those analysed by Mass [45]. Regarding photo-physiological traits, recent findings [15, 16] show increased chlorophyll concentrations in coral holobionts occurring in mesophotic depths. Increased concentrations in antenna pigments per photosynthetic units (PSU) have been related to an acclimation mechanism to low-light regimes [71]. In the present study, as well as in previous works [45, 71], no evidences of acclimation mechanisms (*e*.*g*. increased chlorophyll content) in corals occurring in deep habitats were found. It is important to note that the observed lack of variation in chlorophyll concentration between depths could be related to the fact that we have only analysed the Chl *a* concentration, and did not the total chlorophyll concentration, as performed in the previous studies aforementioned.

Marked differences in reef fish abundance, species richness, trophic groups, size classes and groups of interest were observed when comparing shallow and deeper reefs in the present study. For instance, fish abundance was ~2-fold higher and species richness was up to 70% higher in deeper reefs than in shallow ones. Additionally, larger size classes' individuals (> 25 cm) were almost uniquely recorded in deeper habitats for some ecologically and economically important fish groups, such as great herbivores, groupers and snappers. This difference may be strongly tied to anthropogenic stressors. Similar trends of species size were reported by Bellwood [72] in reefs where no fishing activity occurred and this trend could explain the high impacts that generally occur in the shallow areas. Selective fishing at more accessible shallow reefs tends to remove some individuals and species more quickly, thus creating imbalances in these habitats [72]. However, the impact of fishing pressure in target species may be attenuated in deeper reefs, and these regions may therefore be considered as areas of refuge [73, 74, 75].

As previously demonstrated worldwide [30, 76], we argue that a strong historical spearfishing pressure on shallow coral ecosystems, in addition to other impacts on shallow waters (*e*.*g*. pollution) has modified dramatically reef fish communities in northeastern Brazil. It is important mentioning that depth *per se* could not necessarily have an important influence on reef fish communities and the observed patterns could be explained by distance from the coast as well as differences in depth range of some fish species. Additionally, some recent studies have presented arguments against the “depth refuge hypothesis” and demonstrated a high number of trash and coral bleaching on deeper reefs, thus refuting the idea that deeper reefs could act as refugia (Rocha and Pinheiro, personal communication). However, historical global and local data on fisheries and local ecological knowledge (LEK) data has demonstrated that reef fish communities in shallow waters could be much more similar with deeper reefs in the past and that they have severely declined in the last decades [77, 78]. For instance, interviews with local fishermen from the site evaluated in the present study confirmed this trend for many reef fish species, especially for *Scarus trispinosus*, an endemic and endangered parrotfish. This species is virtually extinct locally in shallow reefs, but being recorded at high abundance in the last decades [38].

## Conclusions

The renewed interest in deep reef communities occurs at a time when shallow reefs are undergoing unprecedented changes [79, 80]. It is worth noting the potential for these communities to serve as refugia for critical taxa, such as fish and corals. Also, it is important to note that sources/sinks for shallow-water coral populations is of increasing interest to both coral reef ecologists and managers [81]. The potential for deeper waters to protect species from natural or anthropogenic disturbances is increasingly recognized as pertinent to marine conservation planning and resource management [26, 27, 82];. In this context, we discuss herein the importance of including and surveilling deeper reef as “no take zones” in Brazilian Marine Protected Areas (MPAs), as previously suggested by [83, 84]. The “APA Costa dos Corais” (Coral Coast MPA), our study site, is the largest Brazilian MPA. However, only its shallow reefs are considered as “no take zones”, and the lack of strictly protecting areas in deeper reefs could compromise the ecological resilience of these ecosystems.

## Supporting information

S1 Table Data from benthonic composition—Bentos_OK_21.06.17. (XLS)

S2 Table Data from fish community—Peixes_09.07.2017_-_Copia. (XLS)
